# Association between diabetes mellitus and adhesive capsulitis of shoulder: A 2-sample Mendelian randomization study

**DOI:** 10.1097/MD.0000000000044119

**Published:** 2025-08-29

**Authors:** Wenqiang Li, Jing Lou, Weizhong Huangfu, Dezhi Han

**Affiliations:** a Department of Burn and Plastic Surgery, No. 969 Hospital, Joint Logistics Support Force of the Chinese People’s Liberation Army, Hohhot, Inner Mongolia, PR China; b Department of General Medicine, Affiliated Hospital of Inner Mongolia Medical University, Hohhot, Inner Mongolia Autonomous, PR China.

**Keywords:** adhesive capsulitis of shoulder, diabetes mellitus, genome-wide association study, Mendelian randomization, single-nucleotide polymorphism

## Abstract

The objective of this study was to investigate whether there is a causal relationship between diabetes mellitus and adhesive capsulitis of shoulder (ACS). A 2-sample Mendelian randomization analysis was performed using publicly available genome-wide association study statistics. To determine whether diabetes was causally associated with ACS, 2 datasets on diabetes among Europeans from the genome-wide association studies catalogue and ACS among Europeans from FinnGen were extracted and the data were analyzed using inverse variance-weighted, Mendelian randomization 123456123451231 Egger regression, weighted medians, weighted modes and simple modes. Sensitivity analysis and heterogeneity analysis were then used to assess the stability and reliability of the results. Mendelian randomization analysis showed causality between type 1 diabetes and ACS (odds ratio = 0.97, 95% confidence interval = 0.85–1.11, *P* < .05). No significant causal effect of type 2 diabetes on ACS (odds ratio = 0.97, 95% confidence interval = 0.95–1.13, *P* = .414). The Cochran *Q* test showed only a low level of heterogeneity between the type 1 diabetes and ACS data sets (*Q* = 106.0431, *P* = .045). Not heterogeneous between type 2 diabetes and ACS. Egger-intercept and funnel plots for data did not show multidirectional and asymmetry at the gene level. The study was the first to find a causal association between type 1 diabetes and the risk of developing ACS in European people. No cause-and-effect genetic link was found between type 2 diabetes and shoulder adhesive capsulitis.

## 1. Introduction

Adhesive capsulitis of shoulder (ACS), also known as frozen shoulder, shoulder periarthritis, or obliterative bursitis, is a relatively common condition characterized by pain in the scapulohumeral joint and a restriction of motion, especially external rotation and abduction.^[1–3]^ It is a prevalent condition that has been the subject of numerous investigations, which have found that it affects approximately 2% to 5% of the general population.^[4]^ It predominantly affects women aged 40 to 60 years. Furthermore, a clear association between adhesive capsulitis and diabetes mellitus (DM) has been established.^[5]^ A higher incidence of adhesive capsulitis is evident in diabetes, with a prevalence that is approximately 2 to 4 times higher compared to the general population.^[6]^

DM is classified into 2 main categories: type 1 diabetes mellitus (T1DM) and type 2 diabetes mellitus (T2DM). T1DM is characterized by high blood glucose. T2DM is characterized by insulin resistance.^[7,8]^ The incidence of DM in adults has risen markedly over recent decades on a global scale. DM can inflict damage upon all organs of the body, including the musculoskeletal system, and can also give rise to numerous health complications.^[9,10]^ Consequently, an increasing prevalence of DM could result in an augmented public health burden.^[11]^ A nationwide, population-based cohort study found that the risk of ACS increases in prediabetic subjects and is associated with T2DM status.^[12]^

The association between diabetes and ACS has been the subject of extensive research for many years, yet the nature of this interrelationship remains unclear. The biochemical rationale for the elevated incidence of ACS in diabetic individuals is speculative.^[13]^ The existence of a genetic predisposition for frozen shoulder is also the subject of considerable controversy. Several studies have indicated that elevated glucose concentrations in diabetic patients may result in augmented glycosylation and cross-linking of collagen within the shoulder capsule.^[14,15]^ In a statistical analysis of an anonymous database on diabetes conducted by Tighe et al,^[16]^ it was found that practitioners are at greater risk of developing diabetes and prediabetes in patients presenting with adhesive capsulitis of the shoulder.

Observational studies to infer causation are inherently limited in that they are restricted to known and properly measured confounding factors. Therefore, to assess the causal association between diabetes and shoulder adhesive capsulitis, we used Mendelian randomization (MR), an application of the instrumental variable (IV) method to the analysis of genetic data.^[17,18]^ MR genetic variants are available with advances in genome-wide association studies (GWAS) and high-throughput genomic technologies. The aim of the MR method is to provide causal effect estimates free from bias due to confounding.

## 2. Materials and methods

### 2.1. Study design

**Figure 1.**
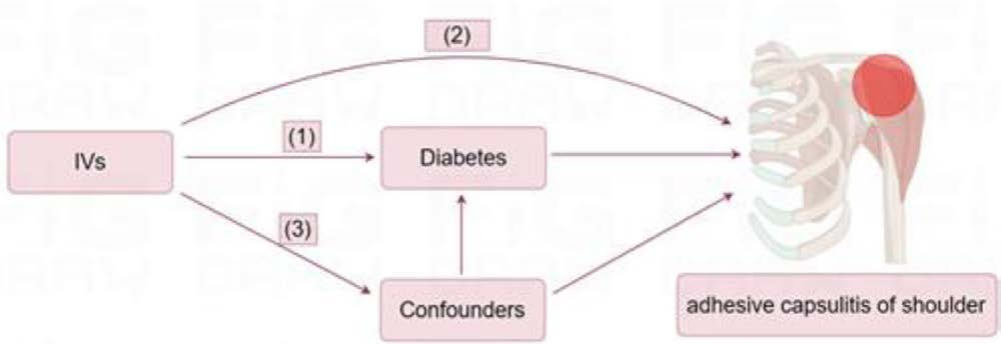
Three key assumptions of the MR study: (1) Relevance: that the choice of IVs has a direct relationship with the exposure of interest; (2) Independence: that the chosen IV is not linked to confounders between exposure and outcome; (3) Confounding: that the chosen IV have no effect on the outcome except through their association with the exposure. IVs = instrumental variables, MR = Mendelian randomization.

This study is a review of secondary data available from existing databases. The 3 key assumptions of MR, as shown in Figure 1, are as follows (1) Relevance: that the choice of IV has a direct relationship with the exposure of interest; (2) Independence: that the chosen IV is not linked to confounders between exposure and outcome; (3) Confounding: that the chosen IV have no effect on the outcome except through their association with the exposure.^[19,20]^ The 2-sample Mendelian randomization (TSMR) analysis was used for the assessment of the causal relationship between diabetes (exposure) and the risk of ACS (outcome).

### 2.2. Data sources and selection of genetic variants

We searched for and collated data from publicly available GWAS. GWAS Catalogue (https://www.ebi.ac.uk/gwas/) and FinnGen (https://www.finngen.f/en) were all consulted in order to conduct this study. The single nucleotide polymorphisms (SNPs) linked to diabetes are derived from a cohort study of T1DMs (18,942 cases and 5,01,638 controls, Chiou et al^[21]^) and a meta-analyzing GWAS in very large samples of T2DMs (62,892 cases and 5,96,424 controls, Xue et al^[22]^) of European ancestry. While significant SNPs associated with ACS were obtained from the FinnGen database (https://www.finngen.fi/fi) of 1,70,583 individuals of European origin (2942 cases and 1,67,641 controls).

### 2.3. Selection of instrumental variables

In order to identify eligible genetic IVs that satisfy the 3 essential MR assumptions depicted in Figure 1, a series of quality control techniques were implemented. From the pooled data sets of the associated GWAS, we first identified SNPs that were strongly associated with T1DM and T2DM (*P* < 5e−08). Next, we checked the SNPs for independent inheritance (<0.001) with no linkage disequilibrium (LD) with each other and with multiple bases between 2 SNPs (kb > 10,000). Subsequently, the allele frequency and LD level were estimated by selecting the European sample data from the 1000 Genomes Project (*R*² = 0.001, window size = 10,000 kb), which was used to estimate the LD between the SNPs and to remove the missing SNPs in the LD reference panel.^[23]^ We also calculated the *F* values of the SNPs to determine the strength of the instruments; an *F* values >10 indicates low bias due to sample overlap.^[24]^

### 2.4. Statistical analysis

**Figure 2.**
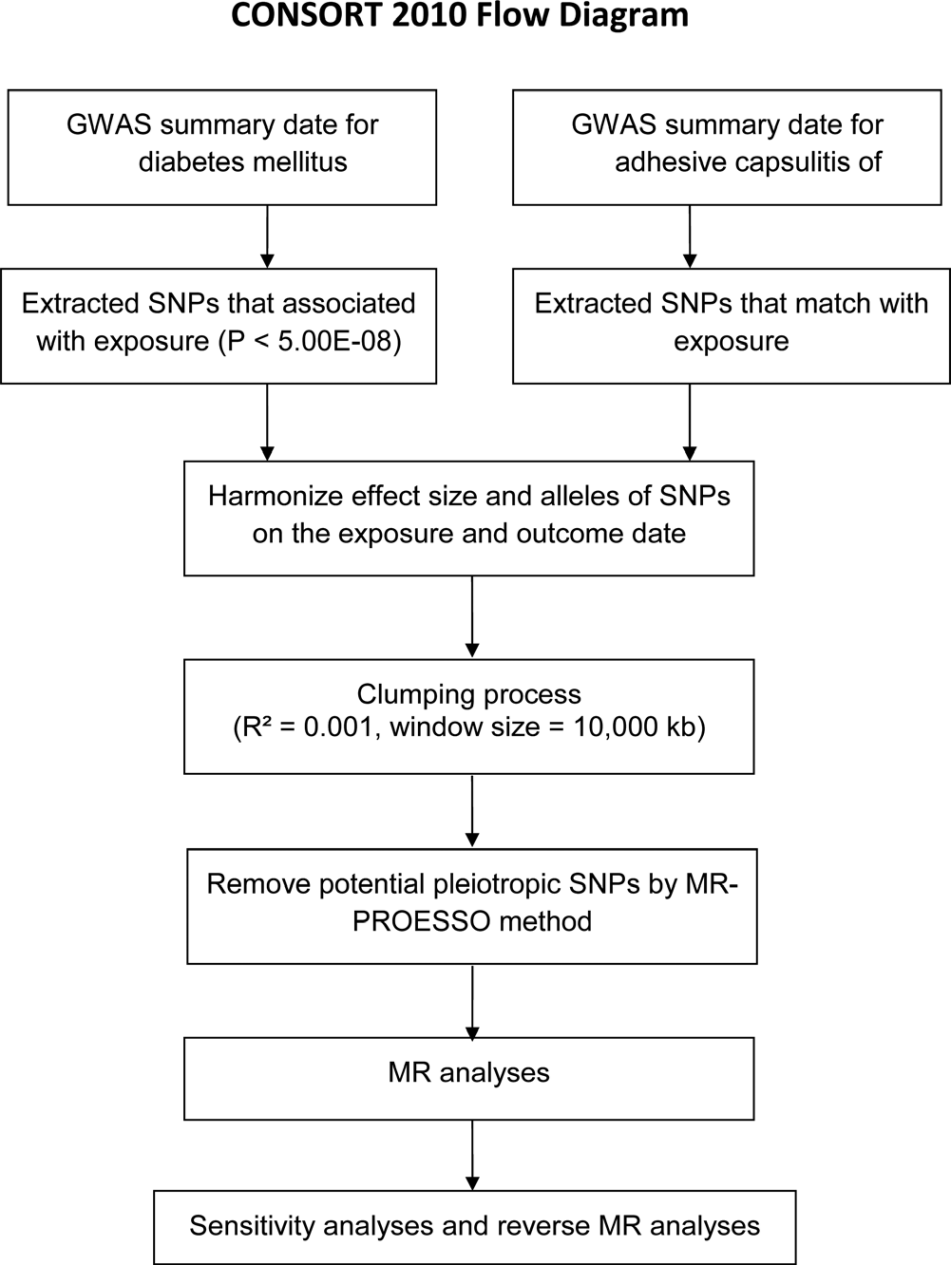
Flow chart of Mendelian randomization analysis. GWAS = genome-wide association studies, MR-PRESSO = Mendelian randomization-pleiotropy residual sum and outliers, SNPs = single nucleotide polymorphisms.

The statistical analysis process is illustrated in Figure 2. In the TSMR analysis, 5 commonly used MR analysis methods were used: inverse-variance weighting (IVW) was selected as the primary analysis for the causal relationship between T2DM and ACS risk. IVW can provide more precise estimates when all IVs are valid. MR-Egger, weighted median, simple mode, and weighted mode methods were also performed. In order to further take into account potential pleiotropy, a number of sensitivity analyses were carried out. The results were subjected to sensitivity analyses such as heterogeneity test and horizontal multiple validity test at the end of the MR analysis. Cochran’s *Q*-test quantifying the heterogeneity of the IVs, with *P* < .05 indicating the presence of heterogeneity, and MR-Egger’s method as a weighted linear regression with intercepts to assess the presence of horizontal pleiotropy among the IVs.^[25,26]^ In addition, leave-one-out sensitivity analyses were performed. In addition, to assess whether the causal effect was significantly influenced by a single SNP, a leave-one-out sensitivity test was performed. All results are presented as odds ratio (OR) and 95% confidence interval (CI), and results were considered statistically significant if *P* < .05.

## 3. Results

### 3.1. Instrumental variables for MR

We performed a TSMR analysis to explore the causal relationship between diabetes and ACS risk. The SNPs were selected from the IEU OpenGWAS database on T1DM (ebi-a-GCST90014023) and T2DM (ebi-a-GCST006867). We selected SNPs strongly associated with diabetes at the genome-wide significance threshold of *P* < 5e−8. Secondly, clumping was carried out using data from the 1000 Genomes Project (*R*^2^ = 0.001, window size = 10,000 kb) in order to estimate LD between SNPs and to eliminate missing SNPs from the LD reference panel. The outcome information for ACS was extracted from the FinnGen database, and the relationship between the above SNPs and the outcome was obtained from the database. Subsequently, the exposure and outcome datasets were merged, resulting in a total of 89 and 118 IVs, respectively, with both outcome and exposure data. Removing the following SNPs for incompatible alleles: rs3135348 and removing the following SNPs for being palindromic with intermediate allele frequencies: rs2303137, rs7936434, rs13234269, rs1758632, rs2058913, rs6494307. The MR pleiotropy residual sum and outlier’s procedure identified and removed a single outlier SNP. The remaining 85 and 114 SNPs constituted the final IV for the exposure. The selection of IVs meets the above criteria (*P* < 5e−8, LD *r*^2^ < 0.001, *F* = *R*^2^ × (N − 2)/(1 − *R*^2^), *F* > 10). It is noteworthy that all of these *F* values were >10, which signifies that 199 IVs were designated as strong IVs in this study. The forest plot illustrates the causal effects of specific SNPs on the risk of depression in patients with ACS. The specific details of the 199 IVs that have been definitively identified are presented in Tables S1 and S2, Supplemental Digital Content, https://links.lww.com/MD/P812.

### 3.2. Causal effects of diabetes and adhesive capsulitis of shoulder

**Figure 3.**
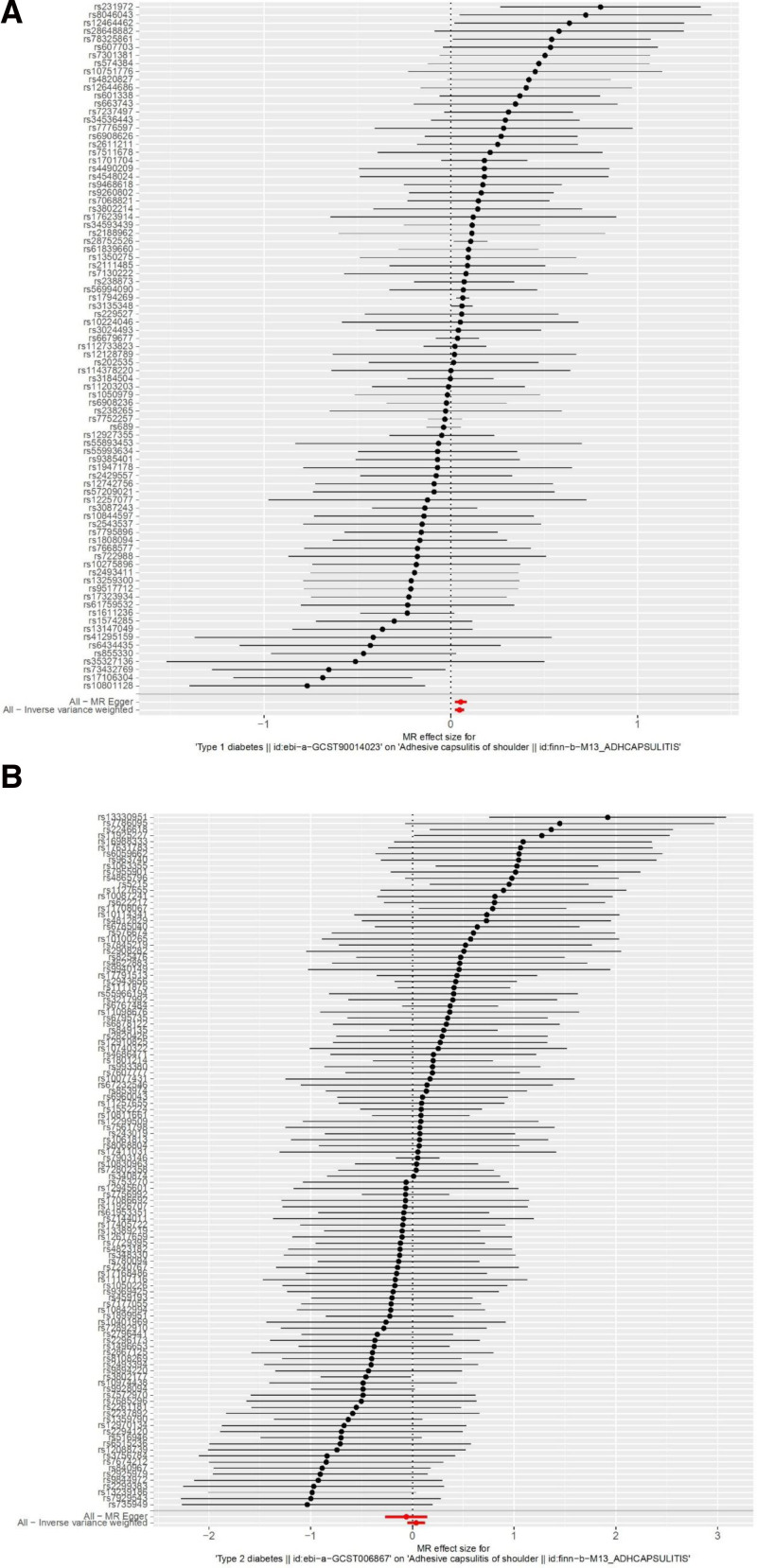
The forest plot displays the causal effect of each SNP in diabetes on T1DM (A); T2DM (B). MR = Mendelian randomization, SNP = single nucleotide polymorphism, T1DM = type 1 diabetes mellitus, T2DM = type 2 diabetes mellitus.

The findings of this study provide genetic evidence supporting the hypothesis that T1DM is a potential risk factor for ACS. However, no causal relationship could be demonstrated between T2DM and ACS. The results of IVW showed a causal relationship between T1DM and an increased risk of ACS IV (β = 0.047, SE = 0.013, *P* < .05, OR = 0.97, 95% CI: 0.85–1.11). With the exception of the imputed model, the results from the weighted median, MR-Egger, and weighted mode were consistent with those of the IVW method. No evidence was present to support a causal relationship between T2DM and ACS by the IVW random effects model method (β = −0.036, SE = 0.044, *P* = .414, OR = 0.97, 95% CI: 0.95–1.13). The comprehensive results of these analytical modalities are presented in a visual format and summarized (Fig. 3 and Table 1).

**Table 1:** Results of Mendelian randomization studies.

Exposure	Method	nSNP	OR	95% CI	*P*-value
T1D	MR-Egger		1.054914	1.0213–1.0897	.001768
	Weighted median		1.063974	1.0319–1.0971	.000072
	Inverse variance weighted	85	1.048393	1.0221–1.0773	.000261
	Simple mode		1.023035	0.9355–1.1188	.619117
	Weighted mode		1.057028	1.0279–1.0870	.000198
T2D	MR-Egger		0.941428	0.7659–1.1572	.567683
	Weighted median		1.049185	0.9063–1.2146	.520328
	Inverse variance weighted	114	1.036868	0.9506–1.1310	.413927
	Simple mode		0.972990	0.7031–1.3465	.869084
	Weighted mode		1.029270	0.8650–1.2247	.745635

CI = confidence interval, MR = Mendelian randomization, OR = odds ratio, SNP = single nucleotide polymorphism.

**Figure 4.**
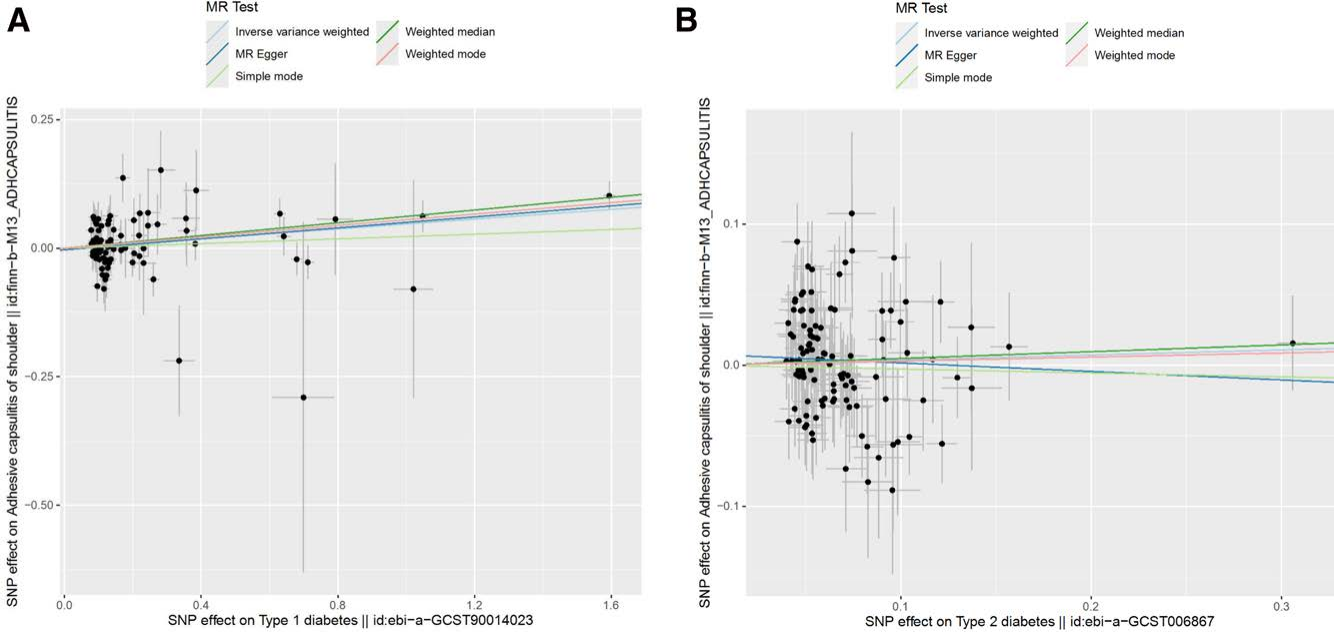
The slope in the scatter plot represents the causal relationship of diabetes on ACS. An upward tilt indicates a positive correlation between diabetes and ACS, while a downward tilt indicates a negative correlation, T1DM (A); T2DM (B). ACS = adhesive capsulitis of shoulder, MR = Mendelian randomization, SNP = single nucleotide polymorphism, T1DM = type 1 diabetes mellitus, T2DM = type 2 diabetes mellitus.

**Figure 5.**
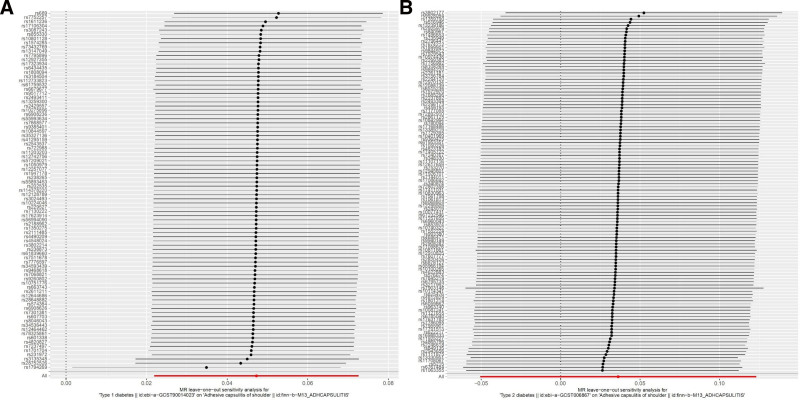
The leave-one-out plot illustrates the impact of individual SNPs on the robustness of the results, T1DM (A); T2DM (B). SNPs = single nucleotide polymorphisms, T1DM = type 1 diabetes mellitus, T2DM = type 2 diabetes mellitus.

**Figure 6.**
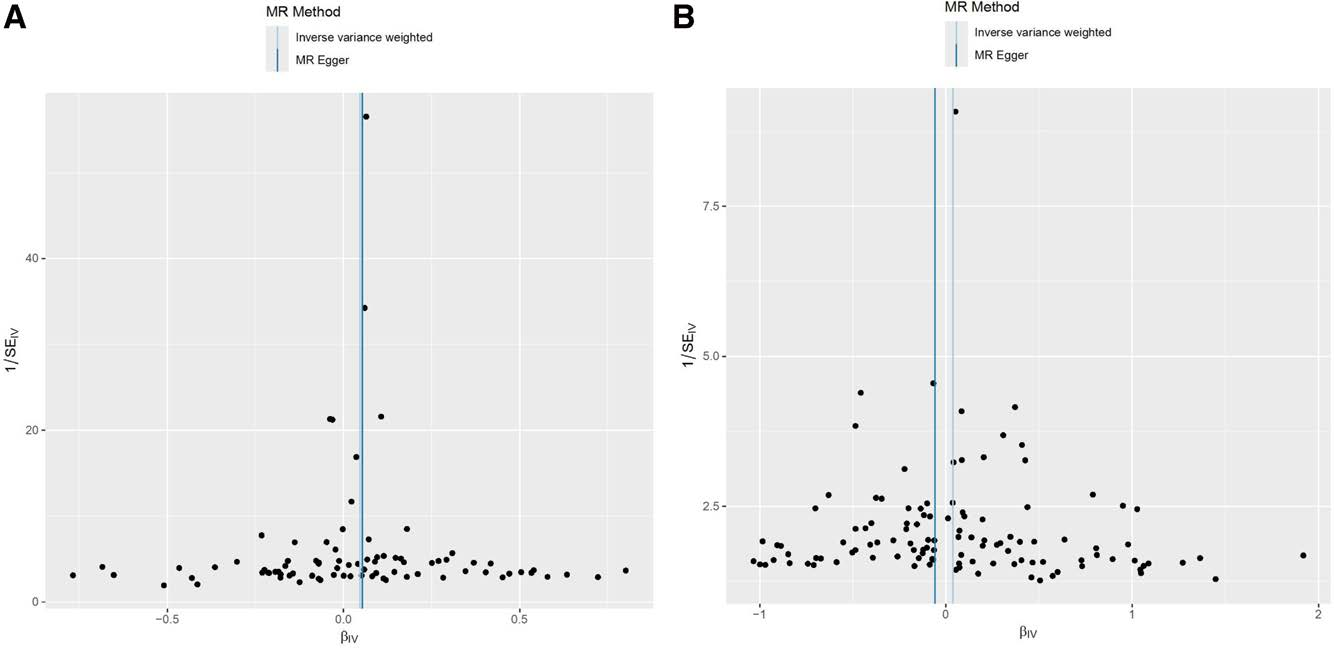
The funnel plot shows the overall heterogeneity of the MR analysis between diabetes and ACS, T1DM (A); T2DM (B). ACS = adhesive capsulitis of shoulder, MR = Mendelian randomization, T1DM = type 1 diabetes mellitus, T2DM = type 2 diabetes mellitus.

Heterogeneity refers to the variability observed in the causal estimates obtained for each SNP. The data indicate significant heterogeneity between T1DM and ACS (*P* = .045, *P* < .05). However, no heterogeneity was found between T2DM and ACS (*P* = .079, *P* > .05). It was acknowledged that heterogeneity may arise from a number of sources, including different analytic platforms, experiments, and population stratification. Random effects modeling was therefore employed to enable MR analysis to be conducted in the presence of heterogeneity. The outcomes demonstrate that the IVs derived from databases did not exert a notable impact on outcomes via pathways other than exposure. As indicated by the Egger-intercept method, this was the case regardless of the specific IVs used (Fig. 4). The leave-one-out sensitivity analysis revealed that the omission of a SNP had only a minor impact on the estimated association of diabetes with the risk of ACS (Fig. 5). The results of the MR-Egger regression test and funnel plot exhibited favorable symmetry and did not indicate any evidence of horizontal pleiotropy (Fig. 6 and Table 2).

**Table 2:** Results of heterogeneity analysis and pleiotropy.

		Heterogeneity test			MR-Egger pleiotropy test			MR-PRESSO global pleiotropy test	
Exposure	nSNP	*Q*	df	*P*-value	Intercept	SE	*P*-value	RSSobs	*P*-value
T1D	85	106.0431	83	.044926	−0.003102	0.005125	.546594	109.9221	.046
T2D	114	133.7544	112	.078871	0.007659	0.007577	.314292	137.2758	.075

MR = Mendelian randomization, MR-PRESSO = Mendelian randomization-pleiotropy residual sum and outliers, SE = standard error, SNP = single nucleotide polymorphism.

## 4. Discussion

This study was the first to evaluate the causal relationships of T1DM and T2DM with risk of ACS from a genetic perspective. Using 2-sample Mendelian analyses, we found that T1DM but not T2DM was a potential risk factor for ACS in the European population. The findings of this study suggest an important role of T1DM in developing ACS, and the prevention and treatment of T1DM may be beneficial for ACS. Despite possible pleiotropy existed between T1DM and ACS, and the MR-Egger test suggesting a significant causal association between T1DM and ACS, it should be noted that MR-Egger is statistically less powerful than IVW. Although heterogeneity exists when T1DM (ebi-a-GCST90014023) is used as an exposure factor, this heterogeneity was allowed because of factors such as population stratification.^[27,28]^

This study is the first to investigate the potential causal relationship between diabetes and ACS using a TSMR approach, which has a great advantage over observational studies because the genetic variants are all measurable and are not affected by the external environment. A study by Kim et al^[12]^ analyzed a large-scale, longitudinal, nationwide, population-based cohort study of 34,71,745 individuals in Korea and reported that the risk of ACS increases in prediabetic subjects and is associated with T2DM status. While our MR analysis showed no causality between T2DM and ACS, but T1DM is a risk factor for ACS, and they are correlated. The discrepancies between our findings and those of previous studies may be attributed to variations in sample size, differing national and regional backgrounds, lifestyle habits, gender, and age. Two other longitudinal population-based follow-up studies^[29,30]^ have reported an association between diabetes and the risk of adhesive capsulitis of the shoulder. Both concluded that the risk of ACS is significantly increased after the development of diabetes, but these studies did not characterize DM. There is some controversy in these studies about whether diabetes is an independent risk factor for ACS. A cross-sectional study by Arkkila et al^[31]^ found that ACS was associated with age in type 1 and type 2 diabetes and with diabetes duration in T1DM.

Most traditional epidemiological studies are case-control designs that fail to clarify causality through ambiguous chronological order. Even in prospective observational studies, it is difficult to avoid the influence of other relevant factors. Randomized controlled trials are widely accepted in the study of causality, but are not easy to perform because they are time-consuming, labor-intensive, and costly.^[32,33]^ By applying the TSMR method, the bias of random assignment of genetic variants at conception can be effectively avoided. Randomized controlled trials were simulated in an observational setting to exclude bias and reveal cause and effect. Second, TSMR also avoids reverse causal effects compared to other observational studies. In this study, TSMR estimates were performed using genetic tools, including T1DM and T2DM of exposure and ACS of outcome summary GWAS data.

This study has a number of strengths. First, to the best of our knowledge, this is the first time the causal relationship between diabetes and ACS from a genetic perspective, avoiding the potential bias of observational studies, and our findings provide a reference for the development of both diseases. Second, TSMR analyses provided non-confounding estimates for the association between diabetes and ACS and all IVs identified were valid, making our findings more reliable. Our study also has some limitations. First, we cannot exclude the possibility of sample overlap. However, our tests indicated that the impact of sample overlap is small.^[18]^ Second, the GWAS data were from populations of European ancestry; therefore, the causal relationship between diabetes and ACS risk should be verified in other populations. Third, heterogeneity was found in the association between T1DM and ACS.

## 5. Conclusion

In conclusion, a significant causal association between T1DM, but not T2DM, and an increased risk of ACS was demonstrated in our MR study. In addition, the associations observed in prior observational studies may have been influenced by unidentified confounding variables. To validate our findings, large-scale GWAS analyses that integrate data and more recent MR investigations of genetic tools are needed.

## Author contributions

**Conceptualization:** Wenqiang Li, Weizhong Huangfu, Dezhi Han.

**Software:** Jing Lou.

**Writing** – **original draft:** Wenqiang Li, Jing Lou.

**Writing** – **review & editing:** Weizhong Huangfu, Dezhi Han.

## Supplementary Material


