# Supplementary material for: Association between diabetes mellitus and adhesive capsulitis of shoulder: A 2-sample Mendelian randomization study
Source: Medicine (Baltimore). 2025 Aug 29;104(35):e44119. doi: 10.1097/MD.0000000000044119 (PMC12401410; doi:10.1097/MD.0000000000044119)
Supplement: Supplementary file 1 [file medi-104-e44119-s001.docx]

| Supplementary Table 1. Detailed information of F-value of SNPs | | | | | | | |
| --- | --- | --- | --- | --- | --- | --- | --- |
| SNP | other_allele | | effect_allele | pval | beta | se | F-value |
| rs348330 | | A | G | 1.86E-09 | -0.0487 | 0.0081 | 69.34148851 |
| rs2820426 | | G | A | 1.30E-12 | 0.0521 | 0.0073 | 81.32412687 |
| rs2493394 | | G | A | 1.15E-10 | 0.073 | 0.0113 | 64.28972837 |
| rs2296173 | | G | A | 7.66E-14 | 0.065 | 0.0087 | 88.90585639 |
| rs340874 | | C | T | 8.41E-18 | 0.0626 | 0.0073 | 121.4466084 |
| rs12088739 | | G | A | 9.79E-12 | -0.0884 | 0.013 | 80.48117628 |
| rs1127655 | | T | C | 2.47E-08 | -0.0438 | 0.0079 | 60.17904454 |
| rs2867125 | | C | T | 4.33E-10 | 0.0601 | 0.0096 | 64.82112489 |
| rs7561798 | | G | A | 2.79E-08 | 0.04 | 0.0072 | 50.28820806 |
| rs780094 | | C | T | 5.16E-21 | 0.0692 | 0.0074 | 143.2343879 |
| rs7572970 | | G | A | 1.39E-11 | 0.059 | 0.0087 | 87.99534232 |
| rs12617659 | | T | C | 2.83E-11 | -0.0685 | 0.0103 | 74.19122072 |
| rs840967 | | A | C | 5.44E-10 | -0.0497 | 0.008 | 74.27384104 |
| rs7607777 | | T | G | 9.40E-28 | -0.137 | 0.0125 | 224.3380328 |
| rs2943656 | | G | A | 6.70E-34 | 0.0902 | 0.0074 | 238.0480871 |
| rs13389219 | | T | C | 2.11E-22 | -0.0722 | 0.0074 | 156.9926239 |
| rs243019 | | C | T | 2.29E-15 | 0.0566 | 0.0071 | 100.1089421 |
| rs6767484 | | G | A | 2.70E-56 | 0.1209 | 0.0076 | 397.320253 |
| rs11708067 | | G | A | 5.93E-29 | -0.0965 | 0.0086 | 213.751857 |
| rs1899951 | | T | C | 1.64E-24 | -0.1118 | 0.0109 | 170.3915384 |
| rs11926707 | | C | T | 1.69E-08 | 0.0463 | 0.0082 | 63.22123931 |
| rs6785040 | | C | T | 1.26E-08 | -0.0633 | 0.0111 | 64.55711734 |
| rs4622883 | | G | A | 3.02E-08 | -0.0435 | 0.0078 | 59.54074328 |
| rs6795735 | | T | C | 1.63E-14 | -0.0558 | 0.0073 | 94.94325926 |
| rs9844972 | | C | G | 1.03E-10 | 0.0956 | 0.0148 | 74.65009201 |
| rs1496653 | | G | A | 2.57E-18 | -0.0769 | 0.0088 | 121.3611039 |
| rs4686471 | | C | T | 4.28E-11 | 0.0534 | 0.0081 | 85.46111802 |
| rs11925227 | | A | G | 2.25E-08 | -0.0534 | 0.0095 | 53.77068555 |
| rs7685296 | | T | C | 2.32E-10 | -0.0511 | 0.0081 | 66.19079313 |
| rs735949 | | C | T | 1.95E-11 | -0.0711 | 0.0106 | 77.17588439 |
| rs11098676 | | C | T | 2.03E-08 | 0.054 | 0.0096 | 61.40799778 |
| rs1801214 | | T | C | 5.52E-34 | 0.0903 | 0.0074 | 247.2019689 |
| rs993380 | | G | A | 4.59E-10 | -0.0507 | 0.0081 | 72.05044465 |
| rs7674212 | | T | G | 6.18E-10 | -0.0465 | 0.0075 | 65.80182559 |
| rs17086692 | | T | G | 2.48E-08 | -0.0467 | 0.0084 | 59.08477012 |
| rs7729395 | | T | C | 1.10E-17 | 0.1373 | 0.016 | 114.8430917 |
| rs4865796 | | A | G | 1.33E-11 | 0.053 | 0.0078 | 75.24598146 |
| rs6878122 | | A | G | 1.19E-12 | -0.0564 | 0.0079 | 86.92273085 |
| rs459193 | | G | A | 8.81E-18 | 0.0711 | 0.0083 | 120.9211963 |
| rs1061813 | | A | G | 3.37E-09 | -0.0429 | 0.0073 | 57.605462 |
| rs10077431 | | A | C | 4.75E-08 | -0.0487 | 0.0089 | 50.33123029 |
| rs622217 | | C | T | 3.13E-10 | -0.0485 | 0.0077 | 73.97703076 |
| rs1063355 | | G | T | 3.72E-19 | 0.0709 | 0.0079 | 151.7990425 |
| rs853974 | | C | T | 7.86E-12 | -0.0601 | 0.0088 | 88.0578077 |
| rs72892910 | | T | G | 6.43E-11 | 0.0648 | 0.0099 | 75.44458235 |
| rs2246618 | | T | C | 1.20E-09 | 0.0513 | 0.0084 | 70.53487816 |
| rs7756992 | | G | A | 6.00E-62 | 0.1297 | 0.0078 | 416.742284 |
| rs9369425 | | A | G | 1.13E-10 | -0.0546 | 0.0085 | 77.58659003 |
| rs1050226 | | G | A | 3.34E-11 | -0.0491 | 0.0074 | 73.25876283 |
| rs3756784 | | G | T | 2.59E-08 | 0.0505 | 0.0091 | 48.57076903 |
| rs849135 | | A | G | 1.04E-43 | -0.0999 | 0.0072 | 315.3940988 |
| rs6960043 | | C | T | 3.61E-19 | 0.064 | 0.0071 | 128.8163597 |
| rs13234269 | | A | T | 6.98E-14 | -0.0583 | 0.0078 | 107.0360056 |
| rs2299383 | | T | C | 1.49E-08 | 0.0412 | 0.0073 | 52.16829018 |
| rs17168486 | | T | C | 2.18E-15 | 0.0742 | 0.0094 | 99.50661911 |
| rs2908282 | | A | G | 4.25E-09 | 0.0552 | 0.0094 | 55.97690068 |
| rs7786095 | | G | A | 9.64E-09 | -0.0743 | 0.0129 | 64.69339123 |
| rs13239186 | | T | C | 2.70E-10 | 0.0539 | 0.0085 | 77.12717576 |
| rs3802177 | | A | G | 2.32E-52 | -0.1217 | 0.008 | 401.9334884 |
| rs2294120 | | G | A | 1.62E-08 | -0.0443 | 0.0079 | 61.28965184 |
| rs17411031 | | G | C | 3.04E-08 | -0.045 | 0.0081 | 49.25431364 |
| rs516946 | | C | T | 3.16E-22 | 0.0824 | 0.0085 | 155.8764641 |
| rs10087241 | | A | G | 2.80E-09 | -0.0475 | 0.008 | 68.46678961 |
| rs7845219 | | C | T | 4.54E-09 | -0.0422 | 0.0072 | 56.0367426 |
| rs10100265 | | C | A | 6.29E-10 | -0.0491 | 0.0079 | 72.18882347 |
| rs2796441 | | A | G | 1.96E-22 | -0.0715 | 0.0073 | 156.6561559 |
| rs3217992 | | T | C | 7.23E-13 | 0.0527 | 0.0073 | 81.52479799 |
| rs10114341 | | C | T | 1.15E-08 | -0.0409 | 0.0072 | 51.90577131 |
| rs10811661 | | C | T | 4.13E-58 | -0.1569 | 0.0098 | 447.3896715 |
| rs1758632 | | G | C | 1.36E-09 | 0.0491 | 0.0081 | 71.27059449 |
| rs10974438 | | C | A | 3.01E-15 | 0.0591 | 0.0075 | 100.2656685 |
| rs17791513 | | G | A | 4.61E-12 | -0.1027 | 0.0148 | 75.61142644 |
| rs1111875 | | T | C | 3.61E-39 | -0.0948 | 0.0072 | 274.2752414 |
| rs7903146 | | T | C | 1.00E-200 | 0.3059 | 0.0077 | 2528.981517 |
| rs11257655 | | T | C | 1.97E-17 | 0.0737 | 0.0087 | 112.2567319 |
| rs753270 | | C | T | 2.70E-11 | 0.0528 | 0.0079 | 85.33235425 |
| rs10740322 | | A | G | 2.11E-08 | 0.0477 | 0.0085 | 61.58797596 |
| rs10830963 | | G | C | 5.85E-30 | 0.0909 | 0.008 | 208.255603 |
| rs67232546 | | T | C | 4.66E-10 | 0.0596 | 0.0096 | 74.00768273 |
| rs1552224 | | C | A | 8.64E-25 | -0.1034 | 0.0101 | 175.8898117 |
| rs2237892 | | T | C | 8.75E-10 | -0.096 | 0.0157 | 67.98406264 |
| rs7929543 | | C | A | 2.20E-09 | 0.0828 | 0.0138 | 65.81639453 |
| rs5215 | | T | C | 2.09E-20 | -0.0678 | 0.0073 | 133.507468 |
| rs7955901 | | T | C | 7.16E-10 | -0.0444 | 0.0072 | 61.25275822 |
| rs11107116 | | T | G | 3.75E-08 | 0.0467 | 0.0085 | 47.06323503 |
| rs61953351 | | T | G | 1.98E-14 | -0.07 | 0.0091 | 115.7427911 |
| rs825476 | | T | C | 6.80E-13 | 0.0524 | 0.0073 | 84.2122806 |
| rs12299509 | | G | A | 2.09E-10 | 0.0467 | 0.0073 | 68.52730879 |
| rs2261181 | | T | C | 9.18E-17 | 0.0985 | 0.0118 | 106.5501534 |
| rs10842994 | | T | C | 1.02E-16 | -0.0755 | 0.0091 | 113.6351965 |
| rs1359790 | | A | G | 2.80E-23 | -0.0796 | 0.008 | 163.4047175 |
| rs576674 | | A | G | 1.79E-11 | -0.0654 | 0.0097 | 75.10232649 |
| rs963740 | | T | A | 2.23E-08 | -0.0479 | 0.0086 | 59.99378098 |
| rs7144011 | | T | G | 1.64E-08 | 0.0482 | 0.0085 | 50.35837141 |
| rs6494307 | | G | C | 1.67E-08 | -0.0443 | 0.0078 | 60.42499953 |
| rs12910825 | | G | A | 2.16E-12 | 0.0517 | 0.0074 | 77.59195061 |
| rs2058913 | | T | A | 3.26E-10 | -0.0491 | 0.0078 | 74.61487197 |
| rs7177055 | | A | G | 2.75E-16 | 0.0647 | 0.0079 | 106.7233721 |
| rs72802358 | | C | G | 1.97E-18 | -0.1168 | 0.0133 | 157.0076682 |
| rs9940149 | | A | G | 9.29E-10 | -0.058 | 0.0095 | 62.12247789 |
| rs9928094 | | G | A | 3.59E-47 | 0.1045 | 0.0072 | 337.673146 |
| rs2925979 | | C | T | 9.06E-12 | -0.0534 | 0.0078 | 75.28835204 |
| rs13330951 | | G | A | 1.54E-08 | -0.0456 | 0.0081 | 65.41773472 |
| rs8068804 | | A | G | 4.41E-14 | 0.0587 | 0.0078 | 95.23559002 |
| rs12945601 | | C | T | 1.72E-09 | -0.048 | 0.008 | 68.78441132 |
| rs17405722 | | A | G | 2.28E-09 | 0.087 | 0.0146 | 65.42857779 |
| rs17631783 | | T | C | 3.95E-08 | -0.0487 | 0.0089 | 57.94026365 |
| rs9894220 | | G | A | 1.52E-13 | -0.0585 | 0.0079 | 105.9008335 |
| rs12970134 | | A | G | 5.31E-12 | 0.0555 | 0.008 | 75.57498238 |
| rs7240767 | | C | T | 2.16E-08 | 0.0451 | 0.0081 | 60.55594559 |
| rs8108269 | | G | T | 3.11E-16 | 0.0644 | 0.0079 | 105.5750041 |
| rs10401969 | | C | T | 4.13E-12 | 0.0921 | 0.0133 | 75.54348472 |
| rs6059662 | | G | A | 1.51E-08 | 0.0446 | 0.0079 | 55.93700969 |
| rs4812829 | | A | G | 2.44E-08 | 0.0532 | 0.0095 | 48.09205393 |
| rs6515236 | | C | A | 3.34E-08 | -0.0504 | 0.0091 | 59.85623668 |
| rs55966194 | | G | C | 2.25E-09 | -0.0526 | 0.0088 | 70.43651598 |
| rs16988333 | | G | A | 9.17E-09 | -0.0745 | 0.013 | 57.45856388 |
| rs4823182 | | G | A | 3.36E-10 | 0.0482 | 0.0077 | 65.23824193 |
| Note: Beta: Estimate coefficient; P-value: P-value from GWAS ; SE: standard error of coefficient estimate. | | | | | | | |

| Supplementary Table 2. Detailed information of F-value of SNPs | | | | | | |
| --- | --- | --- | --- | --- | --- | --- |
| SNP | other_allele | effect_allele | pval | beta | se | F-value |
| rs10751776 | C | A | 2.67E-08 | 0.078145 | 0.01405 | 1593.71988 |
| rs855330 | C | T | 4.89E-11 | 0.111208 | 0.016916 | 2482.975003 |
| rs12128789 | C | T | 3.73E-09 | 0.126969 | 0.021535 | 1930.243938 |
| rs10801128 | G | A | 8.98E-10 | 0.096073 | 0.015681 | 1957.284303 |
| rs17623914 | C | T | 7.97E-09 | -0.134887 | 0.023381 | 1705.922324 |
| rs7511678 | A | G | 3.09E-08 | 0.093508 | 0.01689 | 1612.301261 |
| rs12742756 | G | A | 3.54E-08 | -0.083107 | 0.015077 | 1766.452611 |
| rs6679677 | A | C | 1.00E-200 | 0.64172 | 0.020938 | 47235.13409 |
| rs3024493 | A | C | 7.26E-17 | -0.163855 | 0.019641 | 3667.543074 |
| rs574384 | A | C | 2.20E-08 | -0.133602 | 0.023876 | 1752.320274 |
| rs2493411 | C | T | 1.28E-08 | 0.127063 | 0.022335 | 1933.11368 |
| rs3087243 | A | G | 1.16E-44 | -0.19913 | 0.014202 | 10268.63558 |
| rs4490209 | G | C | 4.55E-08 | -0.084522 | 0.015457 | 1719.371964 |
| rs1881146 | T | A | 4.57E-08 | -0.09517 | 0.017407 | 2028.544734 |
| rs12464462 | G | A | 8.61E-10 | -0.087955 | 0.014341 | 1955.695475 |
| rs2111485 | G | A | 1.05E-18 | 0.127631 | 0.014455 | 4088.44115 |
| rs6434435 | A | G | 1.23E-10 | -0.122856 | 0.019089 | 2120.671516 |
| rs55893453 | G | A | 4.63E-08 | 0.094653 | 0.01732 | 1507.978163 |
| rs28648882 | A | G | 3.03E-08 | 0.098731 | 0.017823 | 1770.136906 |
| rs7668577 | C | A | 7.26E-10 | 0.093652 | 0.015203 | 1967.575089 |
| rs2611211 | T | C | 1.39E-14 | -0.143854 | 0.018689 | 3143.505085 |
| rs12644686 | G | C | 2.44E-08 | -0.10775 | 0.01932 | 1896.997847 |
| rs13147049 | G | A | 8.92E-14 | -0.109522 | 0.014689 | 2889.849453 |
| rs2303137 | T | A | 6.17E-09 | -0.081513 | 0.014025 | 1711.79502 |
| rs114378220 | T | C | 5.11E-09 | 0.177902 | 0.030444 | 2162.59923 |
| rs2188962 | T | C | 1.73E-08 | 0.07946 | 0.014096 | 1587.680337 |
| rs9260802 | G | A | 1.35E-17 | -0.355548 | 0.041635 | 4533.446349 |
| rs7752257 | G | T | 1.00E-200 | -0.679534 | 0.016591 | 112488.0756 |
| rs6908626 | T | G | 6.14E-28 | 0.202923 | 0.01852 | 6003.888253 |
| rs28752526 | G | A | 1.00E-200 | 0.630443 | 0.015912 | 116194.3582 |
| rs4548024 | C | T | 9.95E-09 | -0.095737 | 0.016703 | 1716.12688 |
| rs1050979 | G | A | 5.65E-14 | 0.106196 | 0.014129 | 2949.406976 |
| rs1611236 | A | G | 1.83E-50 | -0.260162 | 0.017415 | 15201.7369 |
| rs3135348 | G | A | 1.00E-200 | 1.04811 | 0.017444 | 613806.2135 |
| rs9468618 | T | C | 5.10E-25 | -0.27213 | 0.026341 | 5849.626708 |
| rs2429557 | A | T | 4.98E-69 | 1.02126 | 0.058158 | 12421.45243 |
| rs9385401 | T | C | 4.59E-16 | 0.12042 | 0.014827 | 3769.597838 |
| rs238873 | G | A | 4.30E-50 | 0.792867 | 0.053276 | 9523.896549 |
| rs73432769 | T | C | 2.34E-13 | 0.335382 | 0.04577 | 2294.185347 |
| rs112733823 | T | C | 7.42E-81 | 0.382619 | 0.020092 | 19257.51042 |
| rs1794269 | T | C | 1.00E-200 | 1.5943 | 0.017743 | -2624860.833 |
| rs6908236 | C | A | 6.31E-32 | 0.166059 | 0.014121 | 7272.184662 |
| rs7795896 | T | C | 1.58E-16 | -0.135435 | 0.016416 | 4102.452061 |
| rs17323934 | G | C | 1.26E-14 | -0.129663 | 0.016818 | 3050.784427 |
| rs10275896 | C | T | 2.60E-13 | -0.121178 | 0.016569 | 2738.363365 |
| rs7776597 | G | A | 1.82E-11 | 0.244413 | 0.036372 | 2457.037846 |
| rs10224046 | G | T | 2.71E-08 | 0.085811 | 0.015436 | 1684.599058 |
| rs13259300 | C | A | 3.28E-10 | -0.092191 | 0.014669 | 2135.986015 |
| rs1947178 | G | A | 1.67E-09 | -0.103267 | 0.017134 | 1835.506938 |
| rs3802214 | C | T | 2.96E-08 | -0.106609 | 0.01923 | 1907.368936 |
| rs1574285 | T | G | 4.27E-19 | -0.126548 | 0.014171 | 4061.743963 |
| rs78325861 | G | C | 2.31E-11 | -0.282082 | 0.042195 | 3123.579924 |
| rs12257077 | T | C | 3.91E-10 | 0.231245 | 0.036955 | 1725.075807 |
| rs7068821 | T | G | 5.07E-24 | -0.165103 | 0.016333 | 5390.827935 |
| rs61839660 | T | C | 5.25E-43 | -0.357441 | 0.026 | 10567.08208 |
| rs41295159 | G | C | 9.11E-15 | -0.699552 | 0.090252 | 4558.911352 |
| rs722988 | C | T | 9.78E-09 | 0.082649 | 0.014412 | 1629.398111 |
| rs7936434 | C | G | 3.58E-08 | 0.076923 | 0.013959 | 1536.707177 |
| rs7130222 | G | T | 1.30E-08 | -0.091985 | 0.016179 | 1884.412095 |
| rs663743 | A | G | 3.50E-11 | -0.099964 | 0.015092 | 2374.575426 |
| rs607703 | T | C | 1.17E-10 | 0.092015 | 0.01428 | 2210.897843 |
| rs689 | T | A | 1.00E-200 | 0.712 | 0.018527 | 129632.3963 |
| rs1701704 | G | T | 4.52E-63 | 0.244048 | 0.014558 | 14276.35016 |
| rs7301381 | C | T | 5.25E-11 | -0.093588 | 0.014258 | 2277.902408 |
| rs10844597 | A | G | 1.20E-10 | -0.089844 | 0.013953 | 2109.247408 |
| rs3184504 | C | T | 1.08E-60 | -0.231498 | 0.014086 | 14269.15522 |
| rs238265 | G | T | 2.08E-09 | -0.090825 | 0.015158 | 1826.975643 |
| rs9517712 | C | T | 1.06E-10 | -0.102063 | 0.015805 | 2089.827285 |
| rs17106304 | G | C | 6.83E-15 | 0.115351 | 0.014812 | 3145.116586 |
| rs1350275 | G | T | 8.86E-10 | -0.093665 | 0.015283 | 1932.600742 |
| rs56994090 | C | T | 3.60E-20 | -0.134255 | 0.014594 | 4643.25746 |
| rs34593439 | A | G | 1.54E-19 | -0.218071 | 0.024119 | 4813.908327 |
| rs12927355 | T | C | 4.41E-41 | -0.203881 | 0.015188 | 9525.490629 |
| rs231972 | C | A | 5.12E-16 | 0.170939 | 0.021081 | 3185.651486 |
| rs55993634 | G | C | 2.29E-19 | 0.219371 | 0.024379 | 3922.023424 |
| rs8046043 | C | G | 2.49E-08 | -0.084587 | 0.015175 | 1781.543456 |
| rs57209021 | T | C | 3.73E-08 | 0.100693 | 0.018297 | 1853.13478 |
| rs61759532 | T | C | 1.91E-10 | 0.118379 | 0.018587 | 2636.259749 |
| rs35327136 | A | C | 3.37E-10 | -0.119196 | 0.018978 | 1954.94291 |
| rs1808094 | C | T | 2.40E-14 | -0.113651 | 0.0149 | 3376.042879 |
| rs7237497 | C | T | 2.71E-32 | -0.220466 | 0.018635 | 6926.716691 |
| rs34536443 | C | G | 1.47E-23 | -0.385331 | 0.038519 | 6396.813478 |
| rs601338 | A | G | 1.20E-18 | 0.127096 | 0.014419 | 4231.247962 |
| rs113374757 | T | C | 1.63E-16 | -0.171277 | 0.020769 | 4200.657932 |
| rs202535 | A | C | 1.79E-14 | -0.141437 | 0.018453 | 2983.199554 |
| rs11203203 | A | G | 1.81E-23 | 0.143803 | 0.014405 | 4938.0822 |
| rs2543537 | T | C | 5.59E-09 | -0.083441 | 0.014316 | 1806.887828 |
| rs4820827 | C | T | 1.01E-19 | -0.129657 | 0.014267 | 4152.301567 |
| rs229527 | A | C | 1.82E-13 | 0.104077 | 0.014138 | 2754.376521 |
| Note: Beta: Estimate coefficient; P-value: P-value from GWAS ; SE: standard error of coefficient estimate. | | | | | | |
